# A preliminary exploration of different coping strategies used by Korean immigrant parents of autistic children in high versus low family quality of life ratings

**DOI:** 10.1177/13623613221133961

**Published:** 2022-11-01

**Authors:** Vanessa C. Fong, Jennifer Shim, Andy Yoon, Bo Sang Lee, Grace Iarocci

**Affiliations:** 1 Simon Fraser University, Canada; 2 Here and Now Community Society, Canada

**Keywords:** autism, community engagement, coping, cross-cultural, family quality of life, interviews, qualitative

## Abstract

The experiences of coping in parents of autistic children have been extensively studied in the literature. While this research has identified both effective and ineffective coping strategies used by caregivers, no studies to date have examined how coping strategies used by parents might be linked to family quality of life outcomes. Furthermore, few studies exist examining both coping strategies and family quality of life in culturally and linguistically diverse populations. Thus, this study aimed to address both limitations. A total of 12 Korean immigrant parents of autistic children, 6 representing the high family quality of life group and 6 representing the low family quality of life group, shared their experiences related to coping and managing stress. Responses fell under three broad categories (problem-focused, emotion-focused, and adjustment-focused) with differences observed when comparing the high versus low family quality of life groups. A better understanding of the link between coping strategies and family quality of life outcomes may help identify effective and culturally sensitive supports for caregivers and families to improve their quality of life and well-being.

**Lay abstract:**

The experiences of coping in parents of autistic children have been extensively studied in the literature. While this research has identified both effective and ineffective coping strategies used by caregivers, no studies to date have examined how coping strategies used by parents might be linked to family quality of life outcomes. Furthermore, few studies exist examining both coping strategies and family quality of life in culturally and linguistically diverse populations. Thus, this study aimed to address both limitations. A total of 12 Korean immigrant parents of autistic children, 6 representing the high family quality of life group and 6 representing the low family quality of life group, shared their experiences related to coping and managing stress. Responses fell under three broad categories (problem-focused, emotion-focused, and adjustment-focused) with differences observed when comparing the high versus low family quality of life groups. A better understanding of the link between coping strategies and family quality of life outcomes may help identify effective and culturally sensitive supports for caregivers and families to improve their quality of life and well-being.

## Coping in parents of autistic children

Cultural beliefs and norms influence childrearing practices from the moment a child is born and have a lasting impact on their development (Meléndez, 2005; Pinderhughes et al., 2000). Parents play a crucial role in scaffolding and supporting their child and all the while maintaining a good quality of life for the family. When a child does not meet developmental norms within a culture, most caregivers are likely to notice delays or deviations from the norm but how they respond to those differences varies greatly depending on one’s culture. However, even within a culture there are differences in how parents cope with differences in development (Bernier et al., 2010; Norbury & Sparks, 2013). Culturally constructed beliefs about the causes of developmental disabilities such as autism not only impact perceptions of autistic individuals and their family members (Grinker & Cho, 2013) but also access to services and supports. Research by Grinker and colleagues (2015) and Kang-Yi et al. (2018) have found that shared beliefs among Koreans that autism is caused by parent mental illness or poor prenatal practices created stigma and impeded caregivers from seeking diagnostic services and supports. Negative attitudes and a poor understanding of autism can create tension among the family and adversely impact parent–child relationships. Although the characteristics and features of autism are similar across different cultures, parenting behaviors, coping strategies, and help-seeking behaviors vary significantly (Fong, Lee, & Iarocci, 2021). Yet we know little about how culture influences childrearing and coping in caregivers of autistic children. Few studies have explored the perspectives of immigrant families living in Canada. In addition to caring for a child with a range of support needs, immigrant families often face language barriers, employment, and housing difficulties and must navigate unfamiliar education, social, and health care systems (Fong, Lee, & Iarocci, 2021; Rivard et al., 2019). A deeper understanding of the role of culture and its impact on childrearing and coping in immigrant families may help in developing services and supports that are better aligned with their needs, values, and preferences, mitigating the risk of poor child and family outcomes.

Though families and caregivers of autistic children report many positive outcomes and enhanced quality of life (Fong, Gardiner, & Iarocci, 2021), various social, systemic, and environmental barriers exist that place them at greater risk for stress and mental health problems (Shakespeare & Watson, 2001). Research has shown that caregivers of autistic children report higher levels of stress compared to parents of typically developing (TD) children and children with other developmental disabilities (Hayes & Watson, 2013). Caregivers of autistic children also report poorer physical and mental health compared to parents of TD children (Khanna et al., 2010).

Consequently, parents must rely on various coping strategies to maintain their emotional and psychological well-being and adapt to meet the demands of daily life. According to Folkman and Lazarus’ transactional theory of stress (Lazarus & Folkman, 1984, p. 141), coping involves “constantly changing cognitive and behavioural efforts to manage external and/or internal demands that are appraised as taxing or exceeding the resources of a person.” Consistent with this theory, coping strategies may involve directly confronting and managing a known stressor (problem-focused coping), regulating the emotions arising from the stressor (emotion-focused coping), or changing the way one thinks about a stressor (adjustment-focused coping).

The experiences of coping in parents of autistic children have been extensively studied in the literature. This body of research has identified both effective and ineffective coping strategies used by caregivers. One study by Cappe et al. (2011), comprising 160 parents of autistic children, found that parents who reported using more emotion-focused coping strategies in their daily lives were more likely to feel nervous, distressed, and guilty. Examples of emotion-focused strategies used included denial, self-blame, and withdrawal. In addition, the use of emotion-focused coping also predicted poorer well-being in their study. However, there is also research supporting the benefits of emotion-focused strategies, specifically positive reappraisal (Lancastle et al., 2022; Ni’matuzahroh et al., 2021; Rayan & Ahmad, 2017). Positive reappraisal is an emotional regulation strategy that involves reframing stressful situations in a positive manner. In a study of 104 parents of autistic children, Rayan and Ahmad (2017) found that positive reappraisal coping was linked to decreased psychological distress, as measured by the Depression, Anxiety, and Stress Scale-21 (Lovibond & Lovibond, 1995). In the Cappe et al. (2011) study, researchers also found that parents who often use problem-focused coping were more likely to report better relationships with their child. Similar conclusions were drawn by Wang and Casillas (2013) in their study of 150 parents of autistic children where it was observed that caregiver use of problem-focused coping was associated with lower stress levels.

A better understanding of coping strategies used by parents of autistic children is needed to identify ways to best support and improve caregiver, child, and family functioning. Despite the breadth of research examining coping strategies in parents of autistic children, few studies exist examining the perspectives and experiences of culturally and linguistically diverse populations.

## Coping in culturally diverse parents of autistic children

Kim and colleagues (2021) explored Korean-American mothers’ constructed cultural identities and experiences parenting an autistic child. Five mothers of autistic children between 7 and 10 years old from the United States were interviewed. Results revealed the daily challenges of caring for an autistic child and the additional challenges related to being newly immigrated. In addition to the financial burden of paying for private therapies and services, parents reported feeling overwhelmed when navigating the special education system. The researchers identified various coping strategies used by parents to effectively manage stress. Having a positive relationship with school staff, identifying improvements in their child, educating themselves about autism and services, and being involved in religious communities appeared to facilitate coping. The importance of relationships with school professionals is highlighted by the finding that Korean mothers of children with developmental disabilities experienced greater stress when in conflict with educators and school staff (Cho & Gannotti, 2005). Spiritual support was also identified as a facilitator for coping for Korean parents of autistic children. Specifically, parents perceived the church community as a key resource for emotional and spiritual support and safe space to facilitate their family’s integration into community life (Kang-Yi et al., 2018).

Although these studies have identified various coping strategies used by Korean parents, we do not understand how coping strategies are related to outcomes such as family quality of life (FQOL). FQOL has recently emerged as an important outcome measure for both researchers and clinicians (Balcells-Balcells et al., 2019). From an applied perspective, assessing FQOL as an outcome measure allows clinicians and professionals to evaluate an individual’s family functioning across a range of areas (e.g. parenting, emotional well-being, physical well-being) and provide recommendations for how best to support a specific family. One assessment tool for measuring FQOL is the Beach Center FQOL Scale (Park et al., 2003). The Beach Center FQQL Scale is a self-report survey comprising 25 items assessing satisfaction across five domains: Family Interaction, Parenting, Emotional Well-Being, Physical and Material Well-Being, and Disability-Related Support. There is strong evidence supporting the validity and reliability of the Beach Center FQOL Scale (Park et al., 2003; Summers et al., 2005; Van Beurden, 2011), and it has also been shown to be user-friendly and easy to administer (Rillotta et al., 2010).

A better understanding of how parents cope, specifically those from culturally and linguistically diverse backgrounds, may help shed light on ways to improve services and supports delivered to underserved populations. Furthermore, understanding how these coping strategies might be linked to FQOL outcomes is critical in terms of how we engage families in optimizing their family well-being. Thus, the aim of this article was to compare coping strategies used by families who reported high versus low QOL.

## Methods

### Participants

Purposive sampling from a list provided by the community partner was used to identify 25 participants meeting inclusion criteria. Inclusion criteria specified that parents had a child diagnosed with autism spectrum disorder (ASD) and that they be of Korean racial/ethnic background. Once the scores on the Beach Center FQOL Scale were computed for all 25 parents, participants were then divided into the highest quartile (top 25%) and lowest quartile (bottom 25%) based on their overall average Beach Center FQOL score. Data for a total of 12 participants, 6 representing the low FQOL group and 6 representing the high FQOL groups were analyzed in this study.

The majority of interviewees were mothers (91.7%). Although this sample is too small to conduct chi-square analysis, the two groups were comparable across most demographic factors. See Table 1 for family and Table 2 for child demographic information across the high and low FQOL groups. The child’s average age was 20.17 years (SD = 9.6) and 17.17 years (SD = 8.0), for the high and low FQOL group, respectively. See Table 3 for the Beach Center FQOL Scale descriptives across the high and low FQOL groups. Children in the low FQOL group appeared to have greater support needs.

**Table 1.** Family demographic characteristics.

Demographic information	High FQOL frequency (%)	Low FQOL frequency (%)
Number of years living in Canada		
5–10	0	1 (16.7)
11–15	3 (50.0)	2 (33.3)
>15	3 (50.0)	3 (50.0)
Respondent relationship to child		
Mother	5 (83.3)	6 (100.0)
Father	1 (16.7)	–
Primary language spoken at home		
English	1 (16.7)	1 (16.7)
Korean	5 (83.3)	5 (83.3)
Primary caregiver age		
30–39	0	1 (16.7)
40–49	2 (33.3)	3 (50.0)
50–59	4 (66.7)	2 (33.3)
Marital status		
Married or common law	5 (83.3)	5 (83.3)
Divorced or separated	1 (16.7)	1 (16.7)
Maternal level of education		
Professional diploma	1 (16.7)	–
Undergraduate degree	3 (50.0)	3 (50.0)
Graduate degree	2 (33.3)	3 (50.0)
Paternal level of education		
Undergraduate degree	3 (50.0)	5 (83.3)
Graduate degree	3 (50.0)	1 (16.7)
Family income		
<$20,000	–	1 (16.7)
$21,000–$49,999	1 (16.7)	1 (16.7)
$50,000–$79,999	3 (50.0)	1 (16.7)
$80,000–$109,999	1 (16.7)	3 (50.0)
$140,000–$169,999	1 (16.7)	–

FQOL: family quality of life.

**Table 2.** Demographic characteristics of focal child with autism.

	High FQOL frequency (%)	Low FQOL frequency (%)
Age of child with autism		
<6	–	1 (16.7)
6–13	2 (33.3)	1 (16.7)
14–18	–	1 (16.7)
>18	4 (66.7)	3 (50.0)
Gender of child with autism		
Male	6 (100.0)	5 (83.3)
Female	–	1 (16.7)
Child’s intellectual functioning		
Low	1 (16.7)	–
Low average	4 (66.7)	4 (66.7)
Average	–	2 (33.3)
High average	1 (16.7)	–
Child’s adaptive functioning		
Low average	4 (66.7)	3 (50.0)
Average	1 (16.7)	2 (33.3)
High average	1 (16.7)	1 (16.7)
Child’s level of behavioral problems		
Mild	2 (33.3)	1 (16.7)
Moderate	3 (50.0)	5 (83.3)
Severe	1 (16.7)	–
Child’s support needs		
Mild	2 (33.3)	1 (16.7)
Moderate	3 (50.0)	3 (50.0)
Severe	1 (16.7)	2 (33.3)

FQOL: family quality of life.

**Table 3.** Scale descriptives.

	High FQOL, M (SD)	Low FQOL, M (SD)	Theoretical range
FQOL domains			
Family interaction	4.53 (0.32)	3.31 (0.69)	1–5
Parenting	4.42 (0.43)	3.19 (0.49)	1–5
Emotional well-being	4.33 (0.61)	2.93 (0.64)	1–5
Physical/material well-being	4.47 (0.37)	3.77 (0.34)	1–5
Disability-related support	4.13 (0.34)	3.33 (0.58)	1–5
Total FQOL score	4.39 (0.33)	3.32 (0.29)	1–5

FQOL: family quality of life; SD: standard deviation.

### Diagnostic confirmation

The inclusion criteria for this study required that the child had received a clinical diagnosis of ASD from a qualified psychiatrist, psychologist, or a pediatrician associated with the provincial government-funded autism assessment network, or a private clinician. Diagnostic assessments were guided by criteria outlined in the *Diagnostic and Statistical Manual of Mental Disorders* (*DSM*-5), which was then verified by both the Autism Diagnostic Interview-Revised (ADI-R; Rutter et al., 2008) and Autism Diagnostic Observation Schedule (ADOS; Lord et al., 1999).

Information regarding child characteristics collected from parents, including intellectual functioning, adaptive functioning, behavioral problems, and child level of support needs is provided in Table 2. An abbreviated survey of child functioning (e.g. intellectual functioning, adaptive functioning) was given to parents considering that for many, English was not their first language. Parents were asked to provide ratings from “Mild” to “Very Severe” on various domains found in lengthier surveys such as the Vineland Adaptive Behavior Scales, Second Edition Survey Interview (Vineland-II; Sparrow et al., 1984) and the Nisonger Child Behavior Rating Form (NCBRF; Aman et al., 1996).

### Interview

An interview guide was developed after consultations with our community partner, self-advocates and their families, and other researchers in the field (Gardiner, 2014). The interview questions were provided prior to the interview to allow participants to familiarize themselves and to prepare an answer if they wished. Consistent with the focus of this study, the interview explored coping strategies used by parents by asking them, “What coping strategies did you find useful when you found out about your child’s diagnosis?” Additional prompts and follow-up questions were also included in the guide to obtain additional information. To provide further context, detailed notes were taken for each parent’s tone, body language, and areas of emphasis. After each interview, a contact summary sheet was completed highlighting important aspects of the interview and potential themes emerging from the data.

### Beach Center FQOL Scale

The 25-item Beach Center FQOL Scale (Hoffman et al., 2006) was completed by each participant. The Beach Center FQOL Scale analyzes five dimensions: Family Interaction, Parenting, Emotional Well-Being, Physical/Material Well-Being, and Disability-Related Support. Respondents rate each statement on a 5-point Likert-type scale ranging from “Very Dissatisfied” (1) to “Very Satisfied” (5). Participants’ responses were converted into domain scores by taking the average of each domain items’ ratings (Hoffman et al., 2006; Park et al., 2003). An average of all item ratings resulted in a computed total score, representing an overall satisfaction score. The scale is internally consistent with alpha values ranging from 0.71 to 0.84 for the domain scores and 0.93 for the overall score. The scale has demonstrated concurrent validity with other family outcome scales such as the Family Resources Scale (Dunst & Leet, 1987), the FRAS-ASD (Gardiner et al., 2019), and the Family APGAR (Smilkstein et al., 1982). In addition, there is preliminary evidence demonstrating internal discriminant validity of the five subscales (Chiu et al., 2017), construct validity (Waschl et al., 2019), and test–retest reliability for each subscale, as assessed 3 months apart (Hoffman et al., 2006).

In addition to the Beach Center FQOL Scale, parents completed a demographic form, and an interview, all of which were offered in either Korean or English to accommodate their language needs and preferences. The demographic form asked respondents to provide information about their relationship to the child, age, marital status, maternal and paternal education, family income, and family members most responsible for caring for the autistic child.

### Procedures

Ethics approval was obtained by the Institution’s Research Ethics Board in British Columbia, Canada. The researcher contacted parents who expressed interest in the study and met inclusion criteria and scheduled interviews with those who wished to participate. Each participant was provided $20 CAD for taking part in the study. Accommodation was given to parents for their language preferences. Parents completed a demographic form, along with an interview in either English or Korean, the latter of which was approximately 35 to 60 min in duration.

Interviews in English were conducted and transcribed by the first author. Interviews in Korean were conducted by the community partner (B.S.L.). Two bilingual research assistants fluent in both Korean and English then transcribed the Korean interviews. Once that was complete, transcribed Korean interviews were then translated to English. Transcriptions and translations of Korean interviews were completed independently, and later compared for verification and accuracy.

### Data analysis

All interviews were transcribed verbatim and analyzed using NVivo, a qualitative data analysis software. The researchers involved in data analysis had extensive background and training in qualitative data analysis and experience in the field of developmental disabilities. Data analysis was guided by Charmaz’s (2006) constructivist grounded theory. Consistent with grounded theory, data analysis occurred immediately after each interview. Constructivist grounded methodology comprises three stages of coding: initial, focused, and theoretical coding. Prior to the initial coding stage, each transcript was read several times to become familiar with the overall content. Important phrases were highlighted and analytic memoing was also used during this stage to identify overall impressions or emergent themes in the data. Initial coding involved fracturing and breaking down the data into smaller components to facilitate comparisons both within and across transcripts (Chun Tie et al., 2019). This stage produced a large quantity of codes where meaningful words or phrases were identified and labeled. The next stage, focused coding, builds on the previous stage but involves more abstract categories to emerge from the data. Coding during this stage involves drawing connections between codes and compiling frequent codes with similar characteristics into more meaningful, higher-order categories. The final stage, theoretical coding, involves further refining codes from the previous stage and selecting a set of central codes that capture key coping strategies used by parents. Codes that reach this stage of analysis tend to represent the experiences of many parents, reduced into highly conceptual terms. Throughout the entire stage, support for this analysis was drawn from the memos that were previously noted during the interviews.

### Trustworthiness

Trustworthiness is defined as the quality and degree of confidence and trust that can be established in the data and interpretation of the findings (Denzin & Lincoln, 2005). The data and interpretation of the study’s findings were guided by Lincoln and Guba’s (1985) criteria to evaluate trustworthiness. The criteria include credibility, dependability, and transferability. Table 4 shows the ways in which this study addressed and fulfilled each criterion.

**Table 4.** Trustworthiness criteria and procedures.

Criterion	Technique	Description
Credibility	Member Checking	Participant responses and researcher interpretations were verified with the participants to ensure accuracy.
	Prolonged Engagement	This criterion was met through various interactions with the community studied by participating at community events, workshops, and continued research activities.
	Peer Debriefing	Feedback regarding theme development, along with coding, was provided by other researchers and peers not involved in the study.
Dependability	Reflective Journaling	This strategy involved recording all research decisions and justifications, coding instructions and their revisions, and theme development.
	Memos	Notes were taken throughout the interviews regarding the participants’ willingness to engage, along with their nonverbal cues, and any contextual details relevant to the study.
	Audit Trail	This technique involved collecting raw data, creating time- and date-stamped memos, and documenting all revisions to coding and theme development.
Transferability	Thick Description	This criterion was met by providing detailed descriptions and direct quotes from participants exemplifying each theme. Descriptions were provided regarding the participants and settings of the research.

### Theoretical saturation

Theoretical saturation is achieved when collecting new data no longer generates new theoretical insights or reveals novel elements about the topic being studied (Corbin & Strauss, 2015). This study did not have a predetermined threshold for determining saturation; instead, this was decided based on the results of our analysis. For example, codes relating to language barriers and acceptance were occurring more frequently and became repetitive when the last few participants were included. Code saturation was reached at 19 interviews (out of the total 25 participants recruited, not the subsample (*n* = 12) analyzed in this study) based on numerous factors such as code identification (95% of codes were identified) and codebook stability (95% of code identification changes had been made). Although saturation became more apparent toward the end phases of sampling, the researcher remained aware of the risk for moving on too quickly and missing potentially important data. Thus, to confirm that we had reached saturation, another 6 participants were included in the study to reach a total of 25 participants. In addition, the researcher made efforts to review earlier interviews and initial coding stages to make comparisons and examine analytic procedures taken. This process allowed the researcher to identify whether there was repetition in terms of codes, processes, and patterns observed or whether there were sufficient data collected to confirm relationships and theories proposed.

### Community involvement

This study placed great significance on community engagement. Community engagement is when “partners contribute expertise and share decision making and ownership to increase knowledge and understanding of a phenomenon, and integrate that knowledge with interventions, policy advocacy, and social change to improve quality of life for communities and reduce health inequities” (Coombe et al., 2020, p. 553). This study has engaged one community partner (B.S.L.), who is both a parent of an autistic adult and the Executive Director of a not-for-profit Korean organization called Here and Now Society. Here and Now Society was established in 2012 and provides supports and services for Korean individuals with developmental disabilities and their families in addition to information and resources in their own language. Self-funded services provided by this organization include Home Camp and Art Studio, and government-funded services include Community Inclusion and Shared-Living programs. B.S.L.’s role involved developing the research questions, providing feedback on the interview guide, recruitment, translating study documents, co-conducting interviews with participants, analyzing data, and knowledge translation. Adopting this paradigm was especially helpful to this study as it ensured that the research employed methods and interpretations of results that were culturally sensitive and contextually grounded in the lived experience of families. This approach also facilitated the recruitment of a hard-to-reach population.

## Results

Parents were asked in their interviews to discuss how they coped with learning about their child’s diagnosis and how they dealt with stress associated with caregiving in their lives. Responses fell under three broad categories: problem-focused, emotion-focused, and adjustment-focused coping. The distinctions between coping strategies used by high versus low QOL families are summarized in Table 5.

**Table 5.** Comparison of coping strategies between high versus low QOL families.

Category	High FQOL	Low FQOL
Problem-focused	Degree of information-seeking: High	Degree of information-seeking: Medium
	Role of social support: To obtain information, emotional support, practical support, connecting with other families	Role of social support: To obtain advice, practical support (childcare)
	Degree of social support: High, complex social networks	Degree of social support: Low, limited to other family members
Emotion-focused	Importance of their faith and religious beliefs; attending parent support groups	Described being fearful; perceived shame and stigma
Adjustment-focused	Positive reappraisal; focusing on child strengths; managing expectations	More frequently mentioned misdiagnosis; isolation; belief in a “cure”

QOL: quality of life; FQOL: family quality of life.

### Problem-focused coping

Information-seeking was frequently identified as important for facilitating understanding and coping for parents in both the high and low FQOL groups. For example, both groups mentioned searching online for informational resources to learn what autism was and how to support their child. However, differences emerged in the degree to which parents from each group engaged in this. For example, parents in the high FQOL group expressed greater urgency and time dedicated to educating themselves (*n* = 5). Parents in this group often mentioned reaching out more expansively in their networks to obtain information and resources. Two parents in the high FQOL group shared,

For me, I tried to find EVERY resource that I can find. And I think that was a very helpful approach.

Study the system. Know the system. Know who to contact and when to contact. And it doesn’t have to be perfect but try to get as much information as possible.

Several parents (*n* = 4) elaborated that information-seeking not only allowed them to better understand how to support their child but also helped them identify the causes of autism reducing self-blame and guilt. It was not uncommon for parents to initially believe that autism was caused by poor parenting skills or prenatal practices. Problem-focused strategies such as obtaining information about the causes and characteristics of autism helped alleviate feelings of shame, guilt, and blame:

Knowledge eliminates those kinds of guilty feelings. Knowledge is important. Truth liberates you.

After he got diagnosed, we understood that this was his condition and he’s not intentionally acting out or being silly or anything like that.

Another defining characteristic of parents from the high FQOL group that appeared to facilitate coping and improve well-being related to the proactive nature of their information- and support-seeking behaviors (*n* = 3). This was emphasized by one parent who suggested,

When service providers provide you with options or services don’t just accept them as they come but be proactive and seek information you need beyond what the service providers ask you to do or provide for you.

In contrast, the low FQOL group often expressed finding information more passively, by “luck,” or having teachers tell them which websites to visit (*n* = 3). Families from this group more frequently reported barriers in obtaining information. Language and communication barriers were most often cited by these families:

I have language issues and I didn’t know what was available so I couldn’t even approach anyone.

There are probably more benefits and services if we have fluent English and no language barrier so we can fight for these. There are probably more services that we can obtain but we have a language barrier and we have a different culture right? That’s the biggest barrier to getting more services. They never told us [about some of these services] before we demanded it. That’s the problem.

The importance of social support and connecting with other parents was emphasized by both the high and low FQOL groups. For example, it was common for parents from both groups to mention meeting other families of children with disabilities through their church or parent support groups. However, the motivations for seeking these supports differentiated the two groups. For example, parents in the high FQOL group (*n* = 5) often mentioned benefits including obtaining information, emotional support, and practical support (e.g. babysitting). These families also seemed to appreciate the value of connecting with other families in similar circumstances as them. Two parents shared,

Without information, without any connections that is very hard for families to not just get information but also just to live a life.

Even though we don’t have family in Canada we have a pretty strong Korean community, church community. Whenever we need support for our child, our family always has someone to take care of him and some of them are professionals that give us some advice for how to take care of our son. When we have to explain or talk about our son it was pretty easy to say “oh he is special, he has autism” and everyone tries to help him and understand him right away.

In contrast, parents in the low FQOL group often mentioned the need to obtain advice and resources as their main motivations for seeking support (*n* = 4). While there were a few parents who recognized the importance of support groups and connecting with members in their community, limited time and access to childcare, feeling overwhelmed, and transportation issues impeded these families from prioritizing this:

We feel so isolated. I cannot do a lot of stuff with his diagnosis so I cannot have the same relationship with my old friends who have typically developing kids.

It’s very hard to have relationships with other families or making friends is also difficult. We almost blocked out our family and experienced no connection actually or very little connection. My family—we don’t feel community support.

### Emotion-focused coping

Parents in the high FQOL group often mentioned the importance of meeting other families and attending support groups in their community to help manage stress. Parents in this group also frequently emphasized the importance of their faith and religious beliefs in facilitating their coping and improving their well-being (*n* = 5). One parent shared,

The people in church have good will. They are willing to find out some help and give us information that they have. Actually, some members of people in church are willing to be a friend themselves and to be a helper.

On the other hand, parents in the low FQOL group often discussed feeling greater negative emotions around the time their child was diagnosed with autism. Several parents recalled that during this period they experienced feelings of fear, confusion, and a sense of loss (*n* = 4). These emotions appeared to partly stem from having a very literal understanding of disability, where parents believed that their child would not be able to do anything or achieve quality of life. In addition, a sense of loss was felt for missing out on career opportunities, social events, and hobbies. Sacrifices made by family members were frequently mentioned in this group:

My husband worked at a really nice company in Korea but he gave it up and had to make changes instead of fulfilling his dreams. He had to make sacrifices.

Before my son got a diagnosis, we were planning on having more children. But once my son got a diagnosis of autism, my husband and I were so afraid of having another child. So that’s the most negative impact on our family. We had a lot of dreams for our family but we needed to re-route our goals or dreams.

For parents in the low FQOL group, it was not uncommon for them to report emotional upheaval and turmoil before eventually coming to terms with their child’s diagnosis. Parents from this group often described their hope in finding a “cure” or “treatment” for their child, which some recognized was misinformed and potentially negatively impacted their well-being. Two parents shared,

I thought it could be cured later. I thought it’s not permanent. As time went on, I realized this can never be cured. The whole family was so shocked. Actually, my whole life stopped and became different.

It was like the end of the world but meanwhile we were trying to know how we can help our child. It was a very sad and tragic moment.

Feelings of denial were also commonly reported by parents in the low FQOL group. In hindsight, parents saw this as a way of allowing them to preserve a sense of hope for their child and family’s future. For these families, obtaining a diagnosis meant confronting their fears that their child was different. Reflecting on this, a number of parents acknowledged that this was counterproductive and detrimental to their child’s and family’s well-being as it contributed to delays in obtaining services and supports. One parent articulated,

Part of it was my ignorance and not knowing what ASD was. And part of me was still in denial like ‘oh maybe he’s just delayed and his development is delayed.’ When the doctor said “he has autism” and ‘it’s severe’ I didn’t want to agree with her. She said “severe” and that word stuck with me. Her saying “severe autism” was really scary. So I didn’t tell anybody that we got the severe part, we just told them “oh he’s got some mild autism.” I always told others and my parents this because I didn’t want to scare them. And maybe I didn’t want to scare myself. It’s scary when a professional says “oh he’s severe” that word is really scary.

### Adjustment-focused coping

Positive reappraisal, which involves cognitively reframing stressful events so they are less threatening or beneficial, was one adjustment-focused coping strategy commonly used by parents. Although this was used by both groups, the high FQOL group often identified using this strategy earlier on and recognized this as crucial to their personal growth (*n* = 4). For example, parents in the high FQOL frequently described how their experience has helped them be more empathetic, patient, and understanding of others. Other parents described personal growth in terms of learning new skills, expertise, and knowledge about autism motivating them to change career paths:

Recently I got hired by another family as a behavioural interventionist. So now I’m working as a therapist, using all my skills and knowledge about ASD. I think it’s going to help me become a better parent for my son.

The biggest impact has been on my career choice. I’m hoping to pursue a career as a behavioural consultant, so that’s one of the biggest impacts he’s had.

A number of parents in the high FQOL group also mentioned how their child’s diagnosis enriched their lives by bringing their family closer and creating stronger bonds between family members (*n* = 4). Parents from this group also described how their outlook on life had changed over the years and conceptualized their journey as a “blessing.” This is illustrated by the following quotes:

It was a huge change but in a good way that maybe bonded our family together. I thought in some way it’s kind of a disguised blessing.

I started to think about what other values are important for families and changed the priority of my life from valuing promotions and making money, to family and God.

I can say that it was not easy, but I can also say that it was a blessing for us to have my son. Because of my son, this allowed my husband, me, and his sibling to work as a team to support our son. And then whoever has any challenges with my son, we always talk about it together and comfort that person.

In the high FQOL group, parents appeared to provide a balanced perspective on both the strengths and challenges they observed in their autistic child. In contrast to the low FQOL group, families in the high FQOL group frequently discussed the strengths of their child and improvements made over time. For example, two parents described their child as follows:

He’s really innocent, pure, funny, and awkward and there are moments in your life that he brings joy into our family.

He’s doing many things independently. Some of the things we can’t do he is doing. He is currently in college, so we are very happy.

In contrast, parents from the low FQOL group frequently mentioned experiencing other stressors around the time their child received a diagnosis, such as divorce, family separation, and issues with immigration contributing to adjustment difficulties and delays in reaching acceptance (*n* = 3). For example, a few parents in this group shared the painful experience of having their child misdiagnosed either in Canada or Korea. These cumulative negative events impacted the family’s well-being and adjustment capacities creating elevated stress:

It was quite a long process. First, I had to talk to my family doctor and she didn’t agree with me and mentioned my daughter is more likely to have ADHD not ASD. Then my husband didn’t really agree with it.

I noticed that my child had difficulties in Korea. He was diagnosed in Korea but at the time it was a language disability, not autism.

On the other hand, highly stressful life events were not mentioned as frequently among parents in the high FQOL group. In comparison with the low FQOL group, parents from the high FQOL group often perceived greater hope and optimism in their child’s and family’s future (*n* = 3). Two parents reflected on their journey as follows:

It was pretty sad for me and my husband but as time went by, we began thinking that this was a blessing for our child because we are able to get support from the government, school, and everywhere. Yeah, so it was really a good thing.

You shouldn’t worry about the label, instead focus on how the diagnosis can help them be better. Being better is the most important thing.

Comparing the two groups, differences also emerged in the length of time it took to reach acceptance in their family. For the low FQOL group, this process seemed delayed and lengthier in duration:

The hardest part was to get over the grieving period, knowing this is a reality, this is not just in my head. I always thought ‘oh maybe it’s a nightmare and this is a dream and I’m going to be waking up soon’ but this was the reality. Knowing for sure that there’s no turning back he has it, that was hard to accept. It took almost three to four years to accept.

However, one parent from the high FQOL group acknowledged the significance of being mindful and not rushing through the coping process of coming to terms with the child’s diagnosis. Rather, this parent advised that caregivers reach out to their community and other families for emotional support and guidance:

In my case I needed some time to accept the entire situation. Parents shouldn’t be rushed or hurried to accept the situation. Instead, take your time and meet people who can share your situation with you and you can get support from them. If we open our eyes, we can find a lot of families who are in the same situation where we can share and get support from them. Just don’t stay at home by yourself.

## Discussion

This study compared coping strategies between Korean immigrant parents of autistic children who rated either high or low on FQOL. Three types of coping strategies were identified by parents in their interviews: problem-focused, emotion-focused, and adjustment-focused. For parents in the high FQOL group, problem-focused coping strategies, specifically information-seeking, were frequently discussed wherein parents described actively and persistently searching for resources and information. For these families, it was also common for them to emphasize the importance of making connections with other parents and families in their community. Most of these parents belonged to parent support groups. This is consistent with previous research demonstrating the positive impact that involvement in the community has on parents raising children with disabilities (Cho & Gannotti, 2005). In this study, benefits from participating in the community included gaining information, resources, as well as practical and emotional support from other parents in similar circumstances. Consistent with other studies (Cho et al., 2000; Choi et al., 2017), parents in the low FQOL group often reported language barriers and difficulties accessing information. However, this study extends these findings by linking these challenges specifically to FQOL. Caregivers in this low FQOL group also commonly articulated feelings of isolation and a lack of community and social support.

Both high and low FQOL groups discussed using emotion-focused coping strategies. Parents from the high FQOL group frequently mentioned the importance of their faith and religious beliefs in helping them cope and manage negative emotions. Current findings are also in line with previous research demonstrating the significant role of religious communities in terms of outreach and providing emotional support for Korean families of children with disabilities (Cho et al., 2000; Grinker et al., 2015; Kang-Yi et al., 2018; Kim et al., 2021). In contrast, parents in the low FQOL group often expressed having less support and experiencing elevated levels of negative emotions such as fear, confusion, and loss. In addition, parents in this group often discussed experiencing greater turmoil, denial, stigma, and emotional upheaval in their process toward accepting their child’s diagnosis. This is consistent with previous research documenting the role of cultural factors such as shame and stigma in impeding acceptance and adjustment in families (Cho et al., 2000; Kang-Yi et al., 2018; Kim et al., 2021). For parents in the low FQOL group, it was not uncommon for them to recall disagreements with their spouse in terms of parenting styles and expectations for their child contributing to elevated levels of stress.

Consistent with previous research (Kang-Yi et al., 2018), the present findings highlight the importance of addressing cultural beliefs about autism and stigma as this appears to be associated with FQOL. Indeed, parents in this study recognized the risks of social isolation and that Korean families may be particularly vulnerable to social exclusion. This can have a negative impact on all family members and may hinder access to early interventions and supports. The current findings also concur with previous recommendations (Grinker et al., 2015; Kang-Yi et al., 2018) that religious communities may be a more effective, culturally safe, and accepting place to provide education and supports for families of children with disabilities.

Differences in the use of adjustment-focused strategies also emerged between the high and low FQOL groups. Although parents in the low FQOL group described efforts to reframe their child’s disability in positive terms recognizing improvements within the child, parents in the high FQOL often tended to elaborate more and identify a greater number of strengths across multiple domains. In addition, families in the high FQOL group frequently highlighted improvements in the lives of all family members and not just the child with disability. For example, parents in the high FQOL group identified improvements in the parent–child relationships and greater family closeness. Similarly, other research has demonstrated closer bonds between spouses and improvements in family interactions (Cho et al., 2000; Kim et al., 2021). Parents in the high FQOL group often described the importance of accepting their child’s diagnosis and setting realistic expectations for their child and family. Rather than viewing the diagnosis as something that should be stigmatized, parents in the high FQOL group often made efforts to reframe their child’s diagnosis as a way to get their child supports and services to improve their family’s quality of life. Similar conclusions were made by Hwang and Charnley (2010) where researchers found that reframing strategies used by siblings of autistic individuals were linked to better outcomes for the autistic child and their family. Several parents also shared the value of advocacy and how mentoring other parents has brought joy and fulfillment in their lives. It appeared that by sharing their insights and lessons learned with other parents this helped facilitate coping and acceptance.

This study has also demonstrated the benefits and feasibility of adopting a community engaged approach for exploring coping strategies and FQOL outcomes in a linguistically and culturally diverse community. Lessons learned from using this approach include the importance of co-design and engaging community members early on. Collaborating with stakeholders, including autistic individuals and their families, service providers, policymakers, and researchers, at the initial stages of a study not only elevates its urgency but also increases its relevance to the community. In addition, to facilitate creating an equal partnership and shared ownership in the research, it is crucial to address power imbalances and anticipate barriers to participation that ultimately impact meaningful engagement.

## Limitations

Several limitations warrant caution when interpreting the findings of this study. The first was that the majority of participants included in the study were mothers. Only a small portion of fathers took part in the study and, therefore, their perspectives on FQOL and coping remain under-represented. Future research should include the perspectives of other family members such as siblings and grandparents to reflect other diverse caregiving situations.

Although the results demonstrate the utility of the Beach Center FQOL Scale in distinguishing the experiences of families categorized as high versus low, the findings must also be interpreted with caution. As a group, combining both high and low QOL families, scores on the Beach Center FQOL Scale were higher in the current sample (M = 3.86; SD = 0.64) compared to previous studies. For example, a study by Gardiner and Iarocci (2015) found an average overall FQOL score of 3.62 (SD = 0.63) in their study of 84 caregivers of children and adolescents between 6 and 18 years. In addition, in another study by Hsiao (2018) of 236 caregivers of autistic children in the United States, mean total FQOL scores reported were 3.50 (SD = 0.76). It is possible that because parents were recruited through a community organization, their experiences and perspectives may not represent those who have less support and/or are less connected with their communities. Future studies using a larger sample size are needed to confirm the current findings.

Another limitation is that data were collected at only one time point. It is important to recognize that the concept of FQOL fluctuates and changes over time depending on various factors and life events. The age of the child and the available supports and services across development will impact the various outcomes assessed in this study. Thus, a more nuanced understanding may be revealed longitudinally assessing the same families over time.

## Conclusion

This study aimed to compare coping strategies used by families who reported high versus low QOL. Findings revealed that coping strategies fell under three broad categories (problem-focused, emotion-focused, and adjustment-focused) with differences observed when comparing the high versus low FQOL groups. Parents in the high FQOL group frequently identified the importance of educating themselves about autism, acceptance, social support, and connecting with their community. Interventions and outreach may be tailored to address these areas in order to optimize parent and family well-being and quality of life. Interviews with parents also highlighted the resilience and resourcefulness of Korean parents, emphasizing the need to leverage these strengths in the design and implementation of culturally informed services and supports for families. Finally, this study has demonstrated the importance of community engagement and the feasibility of engaging members from underserved communities with lived experience in the design and implementation of the research. Previously, research has relied heavily upon traditional empirical approaches where studies are designed for the community rather than with them. This has contributed to significant gaps in our knowledge and barriers implementing evidence to practice. Partnering with members from underserved communities is a crucial first step in building relationships and trust.
